# Establishment of reference interval for hemoglobin A1C and other hemoglobin subfractions for healthy Saudi adults

**DOI:** 10.1371/journal.pone.0300028

**Published:** 2024-03-25

**Authors:** Anwar Borai, Kiyoshi Ichihara, Suhad Bahijri, Abeer Alsofyani, Mohieldin Elsayid, Haitham Husain, Sultanah Boraie, Naif Sannan, Ziad Kalantan, Majdi Jan, Maha Gassas, Mohammed Harbi, Norah Alrowaili, Mohammed Almohammadi, Hawazen Zarif, Mansour Qurashi

**Affiliations:** 1 King Abdullah International Medical Research Center (KAIMRC), King Saud bin Abdulaziz University for Health Sciences (KSAU-HS), King Abdulaziz Medical City, Ministry of National Guard Health Affairs, Saudi Arabia; 2 Faculty of Health Sciences, Yamaguchi University Graduate School of Medicine, Ube, Japan; 3 Department of Clinical Biochemistry–Faculty of Medicine- King Abdulaziz University, Jeddah, Saudi Arabia; 4 Ministry of Health, Jeddah, Saudi Arabia; Para Federal University, BRAZIL

## Abstract

**Background:**

The establishment of Reference Intervals (RIs) for Hemoglobin A1C and other hemoglobin subfractions (A1A, A1B, F, LA1C, A0) is of utmost importance in screening, diagnosing, and monitoring diabetes and other hemoglobin abnormalities through the application of high-pressure liquid chromatography (HPLC) technique. Because there are no locally established RIs for these parameters, it is essential to establish RIs specific to the Saudi population to accurately diagnose and monitor diabetic individuals and identify abnormal levels in hemoglobin subfractions.

**Methods:**

As part of the IFCC global multicenter study of laboratory reference values, a cross-sectional study was conducted in Saudi Arabia. The study involved recruiting a total of 381 healthy adult subjects (>18 years, BMI 28.3 ± 6 kg/m^2^). Blood samples were analyzed for A1C, biochemical and other immunoassay parameters. The need for RIs based on sex, age, and BMI was determined using the standard deviation ratio (SDR) through a 3-level nested ANOVA.

**Results:**

Based on the threshold of SDR≥0.4, RIs for A1C and other Hb subfractions were not partitioned by sex or BMI, but partitioned by age (<45 & ≥45 years) for A1C, LA1C, A0 and F. Spearman’s correlation between glucose, insulin, and C-peptide showed a positive association with different hemoglobin subtractions of A1B, F, A1C, and LA1C. The RIs were obtained by using the parametric method and the latent abnormal values exclusion (LAVE) principle was applied on A1C.

**Conclusion:**

This study established RIs for A1C and other Hb subfractions for healthy adult Saudis. Age was found to be an important source of variation for most of the parameters including A1C. These findings will enhance the understanding and clinical decision-making concerning A1C and other hemoglobin subfractions. The elevated upper limit of RIs for A1C reflects the high prevalence of diabetes in the Saudi population specially in those with increased age.

## Introduction

Diabetes mellitus (DM) is a global epidemic with increasing prevalence, posing a great challenge to healthcare systems worldwide. In 2021, 537 million adults were affected, with a predicted increase to 643 million by 2030 and 783 million by 2045 [1]. Moreover, it has been estimated in 2021 that 541 million adults suffer from impaired glucose tolerance (IGT) and, hence, are at risk of developing type 2 diabetes (T2DM) at a later stage of life.

The prevalence of dysregulated glucose tolerance has been reported to reach an epidemic status in Saudi Arabia with more than half of the population diagnosed with either prediabetes or diabetes [2]. Measurement of glycated hemoglobin (A1C) to monitor glycemic status has been shown to delay the onset of diabetic complications as highlighted by Diabetes Control and Complications Trial [3].

Hemoglobin consists of different hemoglobin subfractions. In adults, hemoglobin (Hb) is composed of approximately 97% Hb A, 2.5% Hb A2, and 0.5% fetal Hb F. In general, only 6% of Hb A is glycated, whereas 94% of Hb A0 is non-glycated [4]. In the presence of glucose, Hb A undergoes a two-step non-enzymatic reaction to produce unstable form labile-A1C, which is then stabilized by an Amadori rearrangement to form Hb A1C. While, glycated hemoglobin A1A and A1B are formed through non-enzymatic attachment of fructose 1, 6-diphosohate and glucose-6-phosphate, respectively [5, 6]. In addition to this there are abnormal hemoglobins such as hemoglobin S and C, which are increased in sickle cell and hemoglobin C disease respectively. More details about different types of normal hemoglobin subfractions which occasionally eluted with A1C using the high-pressure liquid chromatography (HPLC) method are presented in **Table 1**.

**Table 1:** Normal hemoglobin subfractions eluted with A1C using the HPLC method.

Hb Subfraction	Comment about the subfraction	Conditions associated with subfraction level
A0	• A0 consists of two alpha globin and two beta globin chains, and is the most common form of hemoglobin in healthy adults, making up around 95–98% of total hemoglobin. The remaining 2–5% of hemoglobin in healthy adults is typically made up of other variants, such as Hb A2 and Hb F [23, 24].	• Mutations in A0 genes can lead to the production of abnormal hemoglobin, which can cause various types of hemoglobinopathies, such as sickle cell anemia and thalassemia [23, 24].
A1A & A1B	• A1A, A1B, are all normal variants of hemoglobin that differ in the amino acid sequence of the beta-globin chains. The percentage of A1A and A1B in healthy adults hemoglobin is about 0.5% for each. • A1 also referred to as the “fast hemoglobins”. • ‘‘fast” originates from the observation that these elements have a quicker elution from a cation-exchange column compared to other components. • The fractions were described in the order as eluted from the column: A1A, A1B and A1C, respectively.	A recent study shows that A1B is positively associated with renal function and glycemic status, while A1A does not show such association [25]. • Both A1A & A1B are associated with fetal-type erythropoiesis and renal function [26, 27]. • Previous studies show controversial association with A1C [28, 29]. • A1A can be low in iron deficiency, aiding in predicting coexistent iron deficiency in β-thalassemia traits [30].
A1C	• A1C is formed through the non-enzymatic binding of glucose to the N-terminal valine amino acid of the β-chain within adult Hb A. Because of this irreversible glucose binding A1C called Stable A1C (SA1C). • The percentage of A1C in hemoglobin of healthy adults is about 4.0–6.0% of Hb A. • A1C provides an estimation of the proportion of hemoglobin with glucose attached, serving as an indicator of the average blood glucose level over the preceding 2–3 months.	• The higher the A1C level, the poorer the blood glucose control, and the greater the risk of developing diabetes-related complications [31]. • Certain factors can affect the accuracy of the A1C test, such as hemoglobin variants, iron deficiency anemia, hemoglobinopathies, certain drugs, and other medical conditions [31].
F	• Fetal hemoglobin (F) produced by the developing fetus during pregnancy and considered as the main oxygen carrier hemoglobin in the fetus. • Normally, F levels decrease after birth and A becomes the predominant type of hemoglobin. • The percentage of F in healthy adults is up to 2%.	• In some conditions, F levels may remain elevated in adulthood [32] e.g. Hereditary persistence of fetal hemoglobin (HPFH), sickle cell disease, thalassemia, beta-thalassemia major and myeloprolifterative neoplasms [33]. • Hb F decreased in conditions of β-thalassemia major, Diamond-Blackfan Anemia (DBA) and Aplastic Anemia. [23, 34–38]
LA1C	• Glucose enters the red blood cells and binds to hemoglobin rapidly and reversibly through a nonenzymatic process, giving rise to a Schiff base referred to as labile A1C (LA1C) [39]. • LA1C, also known as pre-A1C or pre-glycohemoglobin. • LA1C is a minor fraction of total glycated hemoglobin and is believed to reflect more recent blood glucose fluctuations than A1C.	• Recent findings are in agreement with the view of LA1C, being a marker of recent average glucose exposure [39] to some acute change in the blood glucose level [40, 41]. • Extended storage of samples before analysis could cause red cells to consume glucose, which could lead to the dissociation of LA1C [42, 43].

A1C is a fraction of Hb A. It is considered as a stable ketoamine form of the hemoglobin complex and used to measure the average plasma glucose levels over the past three months [7]. It is often used as a standard biomarker to predict, diagnose, and monitor prediabetic and diabetic people [8, 9]. By monitoring A1C, healthcare providers can get a sense of how well a person’s blood sugar levels have been controlled over the past few months. This can also help identify any trends or patterns that may be contributing to poor blood sugar control. In 2022, the American Academy of Clinical Endocrinology and the American Diabetes Association adopted A1C levels of ≥ 6.5% as a diagnostic criterion for diabetes, and levels of 5.7–6.4% to diagnose prediabetes [10, 11]. However, this percentage is affected by lifestyle habits, age, gender, BMI, race, and ethnic groups [12–19].

The most common methods used for A1C measurement include immunoturbidimetric, colorimetric, and high-pressure liquid chromatography (HPLC). The method of HPLC is considered to be the reference method since it measures not only A1C but other hemoglobin subfractions as well [20, 21]. Thus, it is highly recommended that each laboratory establishes a reference value database that takes into consideration not the level of A1C only but other hemoglobin subfractions also. This is important in diagnostic laboratories as other hemoglobin subfractions are measured simultaneously with hemoglobin A1C when the method of HPLC is applied. This technique is capable of separating common hemoglobin subfractions like A1A, A1B, F, labile A1C (LA1C), A1C, and A0. Abnormality in the concentration of any hemoglobin subfractions, as in sickle cell anemia or thalassemia, will negatively impact A1C result interpretation, which may lead to inaccurate estimation of past glycemia in such individuals. Therefore, in such a case, the clinician must order an alternative glycated protein, such as fructosamine or glycated albumin [22].

Unlike the A1C, literature about RIs on hemoglobin subfractions for healthy individuals is scarce. Although it is well recognized that RIs may vary from one population to another, it is important that these parameters are established for the Saudi population, which is already known to have a high prevalence of diabetes [44]. In addition to this, consanguineous marriage is very common in Saudi populations. Hence, genetic disorders are also prevalent, including diseases related to abnormal hemoglobin synthesis (hemoglobinopathies), e.g., sickle cell anemia and thalassemia [45].

The Committee on Reference Intervals and Decision Limits (C-RIDL) of the International Federation of Clinical Chemistry and Laboratory Medicine (IFCC) has initiated an international multicenter project to establish appropriate reference intervals (RIs) for each laboratory based on local healthy subjects [46, 47]. Within the scope of this initiative, since 2014, we have started to derive Saudi-based RIs for clinical biochemistry [48], immunoassays [49], hematological parameters [50], and recently free light chains [51].

## Methods and materials

### Subjects’ recruitment and blood sampling

In this prospective, cross-sectional study, harmonized IFCC/C-RIDL protocol was followed for recruitment. Regardless of ethnicity healthy adult Saudi Arabian subjects were recruited. The ethical approval from the Institutional review board (IRB) was obtained from King Abdullah International Medical Research Center (KAIMRC), King Abdulaziz Medical City, Jeddah, Saudi Arabia (Study number RCJ0212-209).

However, the preference for recruitment was given to native residents of the Arabian Peninsula. The current study was conducted in the western region of Saudi Arabia. This region includes two large cities, namely Makkah and Jeddah. Announcement for recruitment to the study was through different communication tools, including direct contact, email invitations, social media, posters, banners, or official invitations. Apparently healthy subjects were recruited from different professions, including governmental and non-governmental employees such as health, education, military, retirement office, etc., and non-employees such as jobless, housewives, and new graduates.

The total recruited subjects of 407 were aged between 18 and 65 years. Blood samples were collected between April 2012 and February 2013. Each participant signed an informed consent form. A questionnaire derived from C-RIDL protocol [47, 52] was filled by each subject. The questionnaire includes information about lifestyle, recent infection, clinical history, genetic diseases, medication and/or supplements. Inclusion and exclusion criteria of the modified C-RIDL protocol were applied as previously described [50]. Twenty-eight subjects were specifically excluded: three for abnormal hemoglobin subfractions, two for sickle cell carrier, and 1 for leukemia. The remaining 22 subjects were excluded due to being diagnosed with diabetes mellitus following blood analysis. The average total age ± SD of 38.4 ±12.4 years (39.3±11.6 years for males, and 37.4±13.1 years for females) and males were 51% of the total recruited subjects. The total mean BMI was 28.3 ± 6 kg/m^2^, for males 28.7±5.7 kg/m^2^ and females 27.8±6.3 kg/m^2^. The recruited subjects with allergic conditions such as rhinitis, atopic dermatitis, or asthma were 4.4% for males and 7.1% for females. Current smokers were 29% males and 6% females.

### Blood collection and handling

Blood collection was drawn as described in the established protocol [47]. Samples were collected between 7 and 10 am after fasting for about 10 hours. Venous puncturing was performed in the phlebotomy area of the Department of Pathology and Laboratory Medicine, King Khalid National Guard Hospital, Jeddah. All subjects were Saudis. Anthropometric measurements including blood pressure, height and weight were performed before blood collection. Four plain and two EDTA tubes with a total volume ~15 ml of blood were collected for testing chemistry, immunoassay, complete blood count (CBC) and glycated hemoglobin (A1C). Hemoglobin subfractions were assessed simultaneously with A1C measurement. After blood collection in EDTA tubes, samples were inverted and mixed gently for eight to ten times and then transported together with the plain tubes to chemistry and hematology sections for analysis within 1 hour of collection.

### Measurements

For A1C analysis, Tosoh G8 automated analyzer was used. The analysis was based on the method of HPLC where a column for cation exchange was used to separate the hemoglobin into six subfractions (components) and printed by the Tosoh G8 instrument as follow; A1A, A1B, F, LA1C, A1C and A0. The subfraction A1C is commonly known as glycated hemoglobin. Hemoglobin subfractions were separated by eluting the hemoglobin through the column using different gradients of buffer concentrations.

For complete blood count (CBC) analysis, Cell-Dyn Sapphire Hematology automated analyzer from Abbott Diagnostic, USA was used. All data related to CBC analysis and outcomes were reported in our recently published article [50]. For uncovering subjects with latent diseases, uric acid (UA), low density lipoprotein- cholesterol (LDL-C), high density lipoprotein- cholesterol (HDL-C), triglycerides (TG), gamma-glutamyl transferase (GGT), alanine aminotransferase (ALT), glucose (GLU), insulin (INSUL) and insulin C-peptide (C-PEP) were measured in the aliquot of sera by chemistry and immunoassay auto-analyzers of Abbott Architect 16000c and 2000i as described in our previous studies [48, 49].

Samples analysis was done in the Department of Pathology and Laboratory Medicine at Khalid National guard Hospital. The department was accredited by the College of American Pathologists (CAP) since 2002. The laboratory applies all necessary requirements for internal and external quality control schemes. Bio-Rad Liquichek and calibration results were within acceptable limits on every day of research samples analysis by using Tosoh G8 and Abbott diagnostic instruments.

### Statistical analyses

#### Source of variation of RVs

Multiple regression analysis (MRA) was performed separately for each sex by setting the RVs of each analyte as an objective variable. As explanatory variables, the following four factors were set: age, BMI, Smoking, and Allergy, which were chosen as relevant in the previous RI study for hematological parameters [50]. The association between the objective and each of explanatory variables was expressed using partial correlation coefficient (rp) that gives values between −1.0 and 1.0. As its effect size, we interpreted 0.2≤|rp|<0.3 as weak, 0.3≤|rp|<0.5 as moderate, and 0.5≤ |rp| as strong in reference to the Cohen’s criteria of strength of correlation [53].

#### Partitioning criteria

The decision on the need for partitioning reference values (RVs) to subgroups according to sex and/or age was based on the principle of standard deviation ratio (SDR). The two- and three-level nested ANOVA was used in combination to calculate the standard deviations for between-sex (SDsex), between-age (SDage), between-BMI (SDbmi), and net-between individual (SDindiv) factors. The standard deviation ratio (SDR) for each factor, represented by SDRsex, SDRage, and SDRbmi, was determined by comparing their respective SDs to the SDindiv. The SDR partitioning threshold level was set up to be 0.40 as described by Ichihara et al., 2008 [54]. ANOVA was used to divide the RVs into different age groups. The SDR may not show true variability between subgroups, especially at the lower or upper RI limits (LL, UL). Therefore, a secondary criterion called bias ratio (BR) to lower limit (BR_LL_) and upper limit (BR_UL_) was used to determine the need to split RV [55, 56].

For example, the following formula used for partitioning of sex:

$$BR_{LL}=\frac{\left|{LL}_{M}/{LL}_{F}\right|}{({UL}_{MF}/{LL}_{MF})/3.92},\;BR_{UL}=\frac{\left|{UL}_{M}/{UL}_{F}\right|}{({UL}_{MF}/{LL}_{MF})/3.92}$$

where the M, F, and MF in the subscripts represent males, females, and males + females, respectively. We used 0.57 for BR as a threshold for partitioning [57].

#### Derivation of RI

In this study to calculate the RIs we used the parametric method that relies on Gaussian transformation of RVs using the two-parameter Box-Cox formula [58] and for the calculation of confidence intervals (CI) of reference limits we used the bootstrap technique with iterative resampling of RVs for 50 times. The averages of lower and upper limits (LL and UL) was adopted as the RI limits and the 90% CI of LL and UL were calculated from the mean and SD of LLs and ULs [52].

The latent abnormal values exclusion method (LAVE) is usually used to reduce the effect of metabolic parameters, which are effective in alleviating the influences of latent diseases of high prevalence such as metabolic syndrome, or iron-deficiency anemia by reference to abnormal results among mutually associated analytes [59–61]. In fact, the LAVE method was very effective in our previous studies of determining chemistry analytes and hematology analytes. To assess the utility of applying the LAVE method, Spearman’s correlation coefficient (rS) was used to investigate the relationships between hemoglobin subfractions with different metabolic parameters as shown in **S1 Table**. The effect size of rS was interpreted in analogy to rp calculated by MRA: i.e., 0.2≤|rS|<0.3 as weak, 0.3≤|rS|<0.5 as moderate, and 0.5≤ |rS| as strong in reference to the Cohen’s criteria [53].

## Results

### Sources of variation

Multiple regression analysis (MRA) in males and females was done independently between RVs of each hemoglobin subfraction and source of variations including age, BMI, smoking (4 levels), and allergy (binary), and results are shown in **Table 2**. The r_p_ value of ≥ |0.2| was considered as significant. An age-related increase of RVs was observed for A1C, LA1C and A1B in both males and females while A0 decreased with age in males (r_p_ = -0.352) and females (r_p_ = -0.385).

**Table 2:** Multiple regression analysis of the associations of age, BMI, smoking and allergy with RVs of hemoglobin subfractions.

**Male**	**n**	**R**	**Age**	**BMI**	**Smoking**	**Allergy**
A1A	190	0.241	0.186	-0.113	0.073	-0.101
A1B	192	0.343	**0.200**	**0.266**	0.014	0.076
F	187	0.163	-0.041	-0.052	0.090	-0.112
LA1C	189	0.277	**0.263**	0.054	-0.016	-0.018
A1C	187	0.447	**0.392**	0.129	-0.095	0.057
A0	180	0.380	**-0.352**	-0.123	-0.036	0.019
**Female**	**n**	**R**	**Age**	**BMI**	**Smoking**	**Allergy**
A1A	168	0.221	-0.089	0.140	-0.086	-0.162
A1B	172	0.515	**0.395**	0.186	0.067	0.016
F	169	0.127	**-0.033**	0.032	0.094	0.086
LA1C	169	0.487	**0.425**	0.076	0.115	-0.083
A1C	173	0.516	**0.388**	0.173	-0.051	-0.158
A0	166	0.519	**-0.385**	**-0.216**	0.030	0.024

BMI, body mass index; Smoking and Allergy, binary variables denoting the status of smoking-habits and allergic diathesis, respectively; rp, standardized partial regression coefficient. We regarded 0.2 ≤ |rp| < 0.30 (highlighted in yellow shade) as slightly significant, while 0.3 ≤ |rp| < 0.5 (in green shade) was moderately significant.

At the same time, the r_p_ value of A1B was increased with BMI in males (r_p_ = 0.266) but not in females (r_p_ = 0.186). The r_p_ value of A0 decreased with BMI in females (r_p_ = -0.216) but not in males (rp = -0.123). As shown in **Table 2**, none of the hemoglobin subfractions showed any association with smoking or allergy in both males and females.

**Fig 1:**
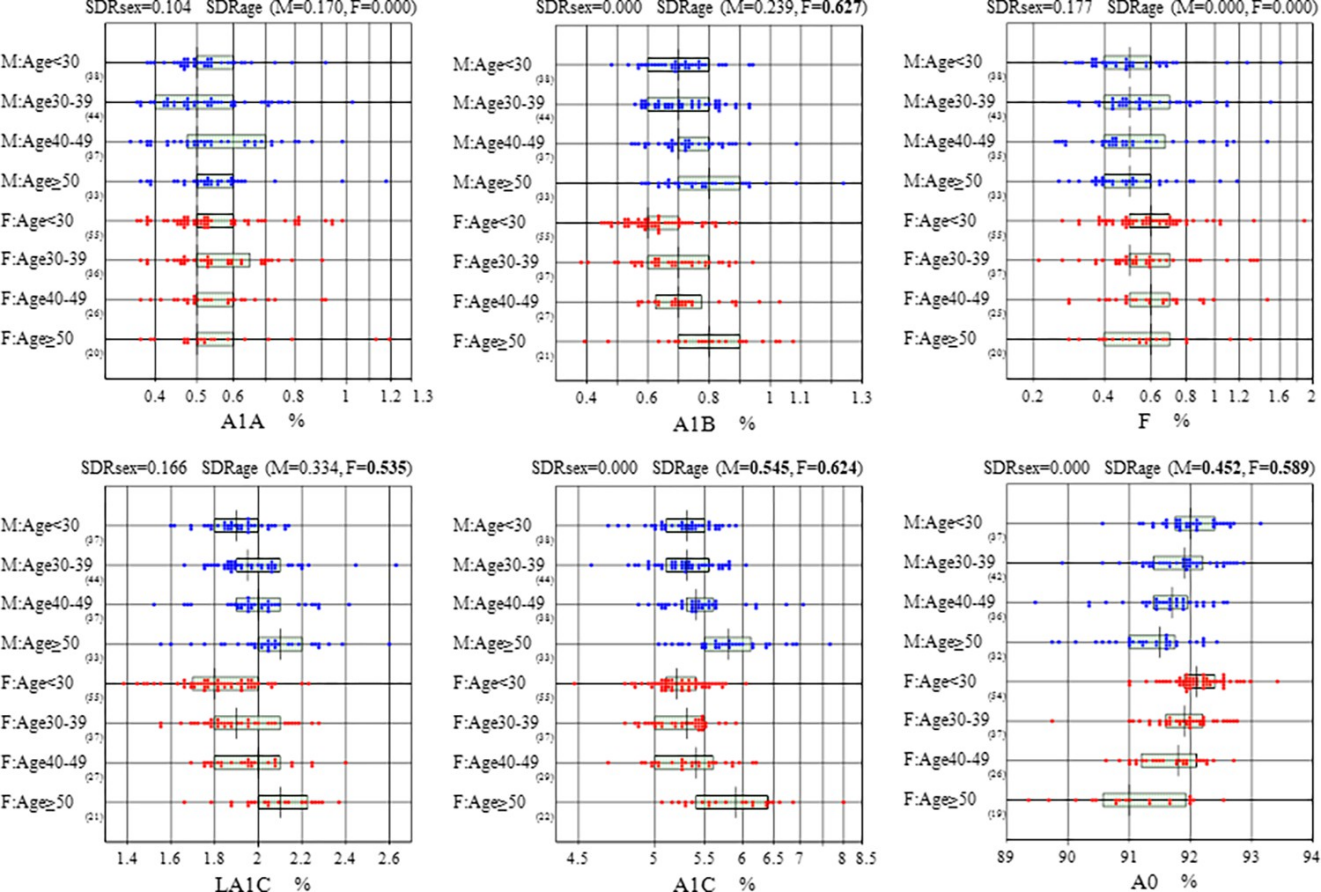
Distributions of RVs for six Hb subfractions partitioned by sex and age categories (<30, 30–39, 40–49 and ≥50 years). RVs of Hb subfraction were partitioned by sex (male: M, female: F) and age-subgroups (∼29, 30–39, 40–49, 50∼). The box in the center of each scattergram indicates the mid 50% range of RVs, and its central vertical bar represents the median. The data size of each subgroup is shown at the right bottom of the age group labels. On top of each graph, values of SD ratio (SDR) for sex and age (SDRsex, SDRage) are shown, which represent the magnitude of between-group differences of RVs after partitioning by sex and age, respectively.

For the estimated SDR, by setting 0.4 as a threshold, sex, age, and BMI were examined as a possible source of variation in the RVs. As a result, age was found to be a significant source of variation for A1B, LA1C, A1C, and A0 **(Table 3A).** This variation was more in females than males for the same parameters **(Table 3B)**. After selecting 45 years (<45 and ≥45), as the age for partitioning the RVs for A1B, LA1C, and A1C were significantly lower in the age group <45 years compared to the older group of ≥45 years. However, this was not noted in the subfractions of hemoglobin A1A and F. This trend of RIs is expected in healthy older subjects when compared to younger subjects for A1C. RIs trend with more detailed age groups (<30, 30–39, 40–49 and ≥50 years) in males and females is shown in **Fig 1**.

**Table 3:** Standard deviation ratios (SDR) for (A) the influence of sex, age, and BMI on different Hb subfractions, and (B) the influence of age on A1C and other Hb subfractions in male and female subjects.

A:					B:		
Analytes	N	SDRsex	SDRage	SDRbmi	Analytes	SDRageM	SDRageF
A1A	365	0.104	0.047	0.104	A1A	0.170	0.000
A1B	371	0.000	**0.466**	0.276	A1B	0.239	**0.627**
F	362	0.177	0.000	0.000	F	0.000	0.000
LA1C	365	0.166	**0.433**	0.000	LA1C	**0.334**	**0.535**
A1C	367	0.000	**0.595**	0.000	A1C	**0.545**	**0.624**
A0	352	0.000	**0.525**	0.261	A0	**0.452**	**0.589**

SDR, standard deviation ratio; SDRsex, between-sex SDR; SDRage, between-age SDR. Between-sex and -age differences were analyzed using Nested ANOVA. An SDR value of ≥ 0.4 indicated the presence of significant between-subgroup differences. SDR values exceeding 0.4 were represented by three distinct colors based on their magnitudes: 0.0 ≤ SDR < 0.4, 0.4 ≤ SDR < 0.6, and 0.6 ≤ SDR.

Sex and BMI did not appear to be significant sources of variation based on SDR values (**Table 3A**). Hence both sex and BMI were not considered when partitioning RVs. By using the Spearman’s correlation coefficient, the association study between hemoglobin subfractions and some of the metabolic parameters, namely gamma-glutamyl transferase (GGT), alanine amino transferase (ALT), Triglycerides (TG), high density lipoprotein- cholesterol (HDL-C), low density lipoprotein-cholesterol (LDL-C) and uric acid (UA), generally showed low levels of correlations (**S1 Table**). The same association study between hemoglobin subfractions and the other metabolic parameters related to diabetes, namely glucose (GLU), insulin (INS), and C-peptide (C-PEP), showed appreciable positive correlation especially with LA1C and A1C, while the hemoglobin subfraction A0 was negatively associated with GLU and C-PEP in both males and females **(S1 Table)**. These findings were interpreted as a rationale for applying LAVE method to reduce the influence of latent metabolic disorders.

### Association between different hemoglobin subfractions

A similar association analysis among hemoglobin subfractions (in both males and females) showed generally high positive associations between A1C, A1B and LA1C **(S2 Table)**. Nevertheless, we did not expect any latent pathological condition of high prevalence related to those analytes. Hence, the LAVE method was not applied to avoid blind trimming of highly correlated peripheral values. Whereas hemoglobin A0 was negatively associated with all other subfractions, especially A1C and LA1C in both males and females, but the phenomenon simply represents complementary changes in percentages.

### Derivation of reference intervals

The RIs were derived by using parametric method based on 90% CI for the upper and lower limits (UL, LL). LAVE procedure was applied by setting simultaneously measured results of glucose, insulin, and C-PEP as reference tests. Detailed RIs with and without applying the LAVE procedure and age restriction based on the bias ratio (BR) cut-off limits are shown in **S3 Table**. We did not consider the requirement for partitioning RVs by gender or BMI based on SDRsex and/or SDRbmi. The SDRage was significant for Hb subfraction F, LA1C, A1C, and A0, and thus their RVs were partitioned by age at 45 years in determining RI: i.e., the boundary was chosen as rational from Fig 1. RIs summary for hemoglobin subfractions are shown in **Table 4**. The source data used for all these analyses are available in **S4 Table.**

**Table 4:** The list of RIs summary with 90% confidence interval adopted without sex consideration.

					Reference intervals by parametric method						
Item	LAVE	Sex	Age	N	90%CI of LL		LL	Me	UL	90%CI of UL	
A1A	(-)	MF	All	366	0.33	0.39	**0.35**	**0.54**	**0.88**	0.84	0.95
A1B	(-)	MF	All	372	0.48	0.51	**0.50**	**0.71**	**1.02**	0.99	1.06
F	(-)	MF	<45	251	0.27	0.30	**0.28**	**0.55**	**1.36**	1.20	1.52
	(-)	MF	≥45	110	0.28	0.31	**0.30**	**0.50**	**1.12**	0.97	1.35
LA1C	(-)	MF	<45	254	1.53	1.63	**1.58**	**1.93**	**2.27**	2.22	2.31
	(-)	MF	≥45	114	1.57	1.70	**1.63**	**2.06**	**2.46**	2.40	2.52
A1C	(+)	MF	<45	215	4.76	4.86	**4.82**	**5.30**	**5.92**	5.82	5.99
	(+)	MF	≥45	100	4.82	5.05	**4.93**	**5.77**	**7.16**	6.87	7.36
A0	(-)	MF	<45	249	90.4	90.8	**90.6**	**91.9**	**92.9**	92.7	92.9
	(-)	MF	≥45	110	89.1	90.1	**89.6**	**91.4**	**92.6**	92.4	92.7

LL, lower limit; UL,upper limit; CI, confidence interval; LAVE, latent abnormal values exclusion method.

### Saudi A1C in comparison to other countries

**Fig 2:**
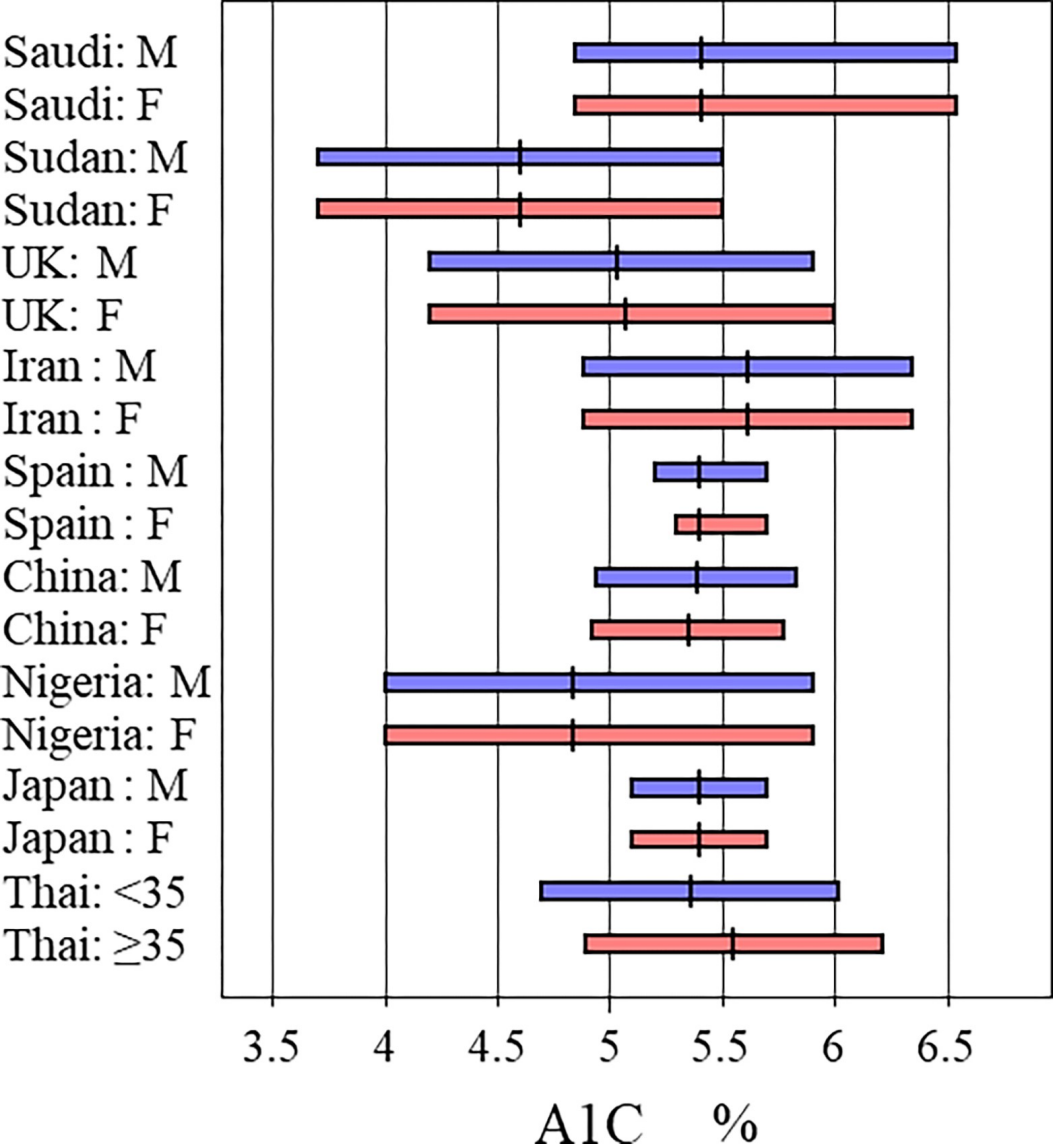
Comparison of RIs for hemoglobin A1C across nine countries.

**Fig 2** Illustrates a comparison between the RIs obtained from Saudi subjects and those from other countries. It seems that our Saudi population involved in this study has the highest upper limit for A1C. Literature about RIs for other hemoglobin subfractions are very limited.

## Discussion

Different hemoglobin subfractions, such as A1, F, A0, and others, can be effectively separated and quantified using the principle of high-pressure liquid chromatography (HPLC). Despite the widespread of HPLC use, there is a limited number of literature available worldwide about reference intervals (RIs) for these hemoglobin subfractions.

It is well recognized that the RIs vary from one laboratory to another depending on different methods and populations. In this study we sought to determine RIs for different hemoglobin subfractions including A1C in adult Saudi population using the HPLC method.

In this study, parametric method (P) was used to obtain RIs for each hemoglobin subfraction. It is of note that this method is known to be effective to the presence of extreme values in the periphery of skewed reference value (RV) distribution [58, 61]. In addition to this, the LAVE method was used to calculate the RIs for hemoglobin A1C only. This method is recognized for its effectiveness in minimizing the impact of highly prevalent subclinical conditions, such as anemia or metabolic syndrome. By the application of both approaches on A1C, the 90% CI of the RI limits became narrower in comparison to nonparametric method (NP) but the differences in the UL and LL were almost negligible **(S3 Table)**. The effect of both parametric and LAVE methods on clinical biochemistry [48], immunoassays [49], and hematological parameters [50]. were more effective in our previous studies.

Compared to other populations, the mean value of hemoglobin A1B in healthy Saudi adults was 0.71%, comparable to the derived mean value of 0.74% from healthy Japanese adults using a similar HPLC method [29]. Similarly, for hemoglobin F, close results were obtained between the mean percentages of hemoglobin F in Kenyan healthy adults of 0.55% [62], and our obtained values for Saudi healthy adults of 0.53%. We also found that the RIs for hemoglobin A subfractions did not vary with sex or BMI which means that both males and females can share the same provided RIs and confirming that the subfractions of hemoglobin A may not be associated to the total quantitative hemoglobin (g/dL) in complete blood count (CBC) which vary greatly between males and females in adult Saudi population as described in our previous study [50].

It is well known that different hemoglobin subfractions are expressed at different stages of human development where hemoglobin F predominates in neonates and declines during the first year of life. At the same time hemoglobin F is a useful parameter for the diagnosis of thalassemia at the early stage of life. Although our study was done on adults, we found that factors of age, still had effect on the percentage of hemoglobin F. This finding is consistent with the previous report about hemoglobin F in African Americans [63].

Although there were not many studies investigated the association between different hemoglobin subfractions but the strong association between LA1C and A1C in our study is consistent with a previous investigation about using LA1C as a potential marker for monitoring rapid occurrence of glycemic disorders [39]. The negative association between A0 with other hemoglobin subfractions in our study is in parallel with the previously obtained outcomes where A0 was found to be negatively associated with A1A, A1C and LA1C [64]. These associations between different hemoglobin subfractions were not investigated enough in previous studies, therefore more investigations are required to explain such associations under different pathological conditions.

Prediabetes is a common health condition characterized by elevated glucose levels that do not reach the diagnostic threshold for diabetes. [65]. The American Diabetic Association (ADA) defines prediabetes as A1C of 5.7% - 6.4% [66] and set the value of 6.5% as the cut off point for the diagnosis of diabetes [67]. Our outcomes showed that the normal mean distribution of A1C in Saudi adults ≤ 45 years is 5.30% with RI upper limit of 5.92% which overlap with the ADA prediabetes in the upper limit. At the same time, the RI upper limit for Saudi subjects with ≥45 years was 7.16%. This limit exceeds the currently used cut off limit for the diagnosis of diabetes of 6.5%, which could cast doubt on the current practice of using HbA1c for diagnosing prediabetes, and diabetes in our Saudi population. Moreover, our results warrant further scrutiny of circulated information regarding the prevalence of type 2 diabetes in Saudi Arabia ranking the country among the top 10 countries, with prevalence rates exceeding the international rates. In 2013, the estimated prevalence of diabetes was 24% [68]. The current prevalence of diabetes in Saudi Arabia is 32.8%. This percentage is expected to increase up to 40.37% in 2025 [69]. Furthermore, these results suggest possible differences in genetic and environmental factors affecting the population of Saudi Arabia. These are supported by the outcomes from previous studies, which show that the underlying pathophysiological mechanisms of the glucose intolerance state leading to diabetes in Arabs may require different strategies to prevent diabetes [70, 71].

The clinical decision limit (CDL) for A1C described in the ADA guideline [72] is widely accepted as relevant for diagnosing diabetes. Nevertheless, this study is still meant to determine RI as a central 95% range of values representing the apparently healthy Saudi population. This was because we have no means of determining the CDL that requires prospective epidemiological study and because we still need to know the actual profile of A1C values in relation to the sex and age of healthy individuals for proper clinical interpretation of A1C results. Besides, we sought to establish RIs for other associated hemoglobin subfractions rather than using RIs from the manufacturers or other populations. The outcomes of this study will help detect any abnormality in hemoglobin subfractions in Saudi subjects while performing routine measurements of the A1C test for diabetes diagnosis.

Our elevated mean values of 5.30% and 5.77% for A1C in our recruited subjects with ages of <45 and ≥45 years, respectively, are consistent with another local study where the mean value was 5.51% for healthy Saudi subjects [73]. Considering our obtained RIs of A1C (4.82–5.92%) for the age of <45 years, and comparing this to ADA definition for prediabetes [66], it seems that many Saudis who are apparently healthy young subjects, but in fact, they can be considered as prediabetic. Hemoglobin A1C usually increased in an elderly subjects [15] and it can happen regardless of the presence of diabetes [74] but the obtained RI for A1C with the mean of 5.77% in old Saudis who seem apparently healthy subjects need further investigations. This outcome is consistent with the previous literature, which have been most likely based on current ADA recommendations, about the very high prevalence of diabetes in elderly Saudi males and females aged 50 to 59 years of 60.2% and 57.1% respectively, and those aged 60 to 69 years of 71.8% and 64.9% respectively [75].

Previous studies show that the diagnosis of prediabetes using A1C criterion associated with a higher diagnostic rate compared to the use of fasting plasma glucose [76, 77]. However, by using the same cutoff values, our outcomes are compatible with the previous studies as we found a higher prevalence of prediabetes in all our subjects by using A1C compared to fasting glucose level (26.0% vs. 20.7%) while for subjects with ≥45 years of age the prevalence increased to 41% vs. 22% respectively. This indicates that the addition of A1C in the screening process for subjects with ≥45 years might improve the prediction of prediabetes and diabetes in the Saudi population. This point is important and may need further extensive and larger cohort studies using biochemical markers as well as clinical outcomes to reach evidence-based guidelines for diagnosing dysglycemia in our Saudi population.

Indeed, our study showed significant variations in the level of A1C of healthy Saudis in comparison with the RIs of international guidelines and other populations. The study results revealed an urgent need to build national guidelines for glycemic parameters for healthy Saudi adults and elderly. Further studies to confirm these results and establish new threshold limits based on the relationship between glucose levels and long-term complications for A1C in Saudis are highly important.

The diagnosis of prediabetes is important, as it is a risk factor for the development of type 2 diabetes. The standard method to identify individuals with prediabetes is to use the oral glucose tolerance test (OGTT). A previous study about Japanese showed that the A1C with the cutoff value of 6.0% is appropriate to identify prediabetes with a diagnostic performance of 83.7% sensitivity and 87.6% specificity in comparison to OGTT [78]. Unfortunately, in our study the method of OGTT was not applied to our recruited subjects, which can be considered as one of the limitations of our study. Therefore, without OGTT establishing a reference interval for A1C can not discriminate reliably between healthy and subjects with dysglycemia, but it can give an indication about the average, minimum, and maximum limits of A1C in individuals who suppose to be healthy in the population and they can be used to know the glycemic status of a population in comparison to other populations.

In summary, the healthy subjects included in this study from Saudi Arabia were carefully selected in accordance with the standardized IFCC/CRIDL protocol. By utilizing both parametric and LAVE methods, the accuracy and reliability of the calculated reference intervals (RI) were enhanced, and the influence of patients with underlying health conditions was minimized. Additional limitation of this study is that it solely recruited healthy subjects from the western region of Saudi Arabia. However, some of the recruited subjects were born in other regions of Saudi Arabia, hence, could be considered as representatives of these other regions. Nevertheless, it is noteworthy that in our previous studies we found no regional differences in the RIs of commonly measured chemical and immunoassay parameters in subjects recruited in the three major regions of Saudi Arabia (Western, Central and Eastern regions) [48, 49].

## Conclusions

Replacing the currently used RIs for different hemoglobin subfractions with the newly determined RIs, which are based on the local population, will presumably reduce the error of interpreting test results measured by the HPLC method especially for those subfractions reflecting hemoglobinpathies. This will contribute to the safety of our diabetic patients’ screening, monitoring, and management. In addition to this, the increased level of A1C in elderly Saudi subjects needs special attention, and further investigations to setup national threshold guidelines and recommendations are required.

## Supporting information

S1 Table Spearman’s correlation coefficients between hemoglobin subfractions and other metabolic related parameters. Metabolic parameters (GGT, ALT, TG, HDL-C, LDL-C and UA), showed weak associations with hemoglobin subfractions in general, while glucose (GLU), insulin (INS) and C-peptide (C-PEP) showed moderate associations with LA1C, A1C and A0 hemoglobin subfractions. Hence, they were used in applying the LAVE procedure to exclude individuals with abnormal glucose metabolism.
(XLSX)

S2 Table Spearman’s correlation coefficients between hemoglobin subfractions. Hemoglobin subfractions (A1A, A1B, F, LA1C, A1C and A0) showed different degree of association between each of them in both males and females.
(XLSX)

S3 Table Full results of RIs determined with/without LAVE, considering sex, and age. (XLSX)

S4 Table Study source data. (XLSX)
